# Comparing Electrolytes in Prestorage Leukocyte-Reduced Packed Cell versus Unfiltered Packed Cell 

**Published:** 2013-07-22

**Authors:** L Fallahi, R Ghiliyan, A Hashemi, A Fatemi, M Saeedi

**Affiliations:** 1 MSc of Biochemistry, Payam nor University, Taft, Yazd, Iran.; 2 Internal Medicine. Hematology, Oncology and Genetics Research Center Shahid Sadoughi University of Medical Sciences and Health Services, Yazd, Iran.; 3 Department of Pediatrics, Hematology, Oncology and Genetics Research Center, Shahid Sadoughi University of Medical Sciences and Health Services, Yazd, Iran.; 4 Blood Transfusion Center, Yazd, Iran.

**Keywords:** Electrolytes, Blood Transfusion, Leukocytes

## Abstract

**Background:**

Blood transfusion is associated with side effects caused by residual leukocytes in blood and blood components. Using leukodepleted blood components can decrease some of these adverse effects. Among the various methods to remove leukocytes in blood components, prestorage leukoreduction has been most efficient, but the evidence of clinical side effects awaits further studies. We evaluated changes of electrolytes in prestorage leukocyte-reduced red blood cells.

**Materials and Methods:**

In this case-control study, one hundred twenty eight packed cells were studied: 64 unfiltered packed cells and 64 prestorage filtered packed cell. Two groups were matched as sex and age. Electrolytes such as Calcium, Sodium, and Potassium of two groups were measured, and compared during preparation.

**Results:**

In this study, mean of Calcium in unfiltered and filtered group were 6.16±1.09 mg/dl and 5.57±2.21 mg/dl, respectively (P-value<0.055). Mean of Sodium in unfiltered group also was 155.91+/-9.51meq/l and in filtered group, 153.05+/-13.21meq/l (P-value<0.163), and mean of Potassium in unfiltered group was 5.01+/-1.72 meq/l and in filtered group, 7.42+/-2.45meq/l (P-value<0.001).

**Conclusion:**

Releasing of Potassium during preparation of prestorage leukoreduction can cause increased Potassium level and hemoglobin concentration changes in prestorage filtered packed cell.

## Introduction

Transfusions of red blood cells are the main treatment for people who have severe hematological disorders. The presence of leucocytes in blood components is responsible for many of the complications associated with blood transfusion such as transmission of cell-associated infectious agents, febrile non-hemolytic transfusion reactions, and refractoriness to platelet transfusion, graft - versus - host disease, generalized immune suppressant, and an increased graft rejection rate of marrow or kidney transplantations (1).

The United States Food and Drug Administration (FDA) allow blood components to be labeled as leukocyte reduced if they contain fewer than 5 ×10^6^leukocytes. The standard in many European countries is less than 1×10^6^ leukocytes per component (2). Commercially available leukocyte removal filters frequently have been used to produce leukocyte depleted blood products. This has been in an effort to decrease transfusion reactions. Leukocyte reduction (LR) can be performed prior to storage, or prior to transfusion at the patient’s bedside. Prestorage leukocyte-reduced red blood cells require special preparation by removing leukocytes (white blood cells) or by filtration shortly after donation. This has performed before storage, as high numbers of leukocytes remaining in a unit of RBCs during the storage process can fragment, deteriorate, and release cytokines (chemicals that affect the inflammatory response). Post storage LR would not be expected to remove these cytokines. Prestorage leukoreduction of red blood cell and platelet products was instituted by the Canadian Blood Services between November 1997 and August 1999 with the aim of preventing the complications of transfusion associated with “passenger leukocytes” (3,4). As a consequence, the use of leukodepleted blood components is widely practiced and recommended but prestorage LR of RBCs resulted in a number of ocular reactions consisting of conjunctival erythema and/or hemorrhage (5, 6). Despite the use of additive solutions, during storage erythrocytes cause some complex structural, functional, metabolic, and electrolytes alterations, which are called storage lesions (7, 8, and 9). These changes destroy the cells irreversibly and ultimately, reduce the function and survival of RBCs following transfusion (10). The most significant change that occurs in electrolytes during storage is the increase of serum K (7, 11). Thus, aim of this study was to evaluate the electrolytes change of prestorage leukoreduction of red blood cell comparing to unfiltered packed cell.

## Materials and Methods

The present research was a case control study. 128 packed RBC evaluated in Blood Transfusion Center, Yazd, Iran. We had two groups: 64 unfiltered packed cells and 64filtered packed cells. There were not differences between donors with respect to age, sex and hemoglobin concentration (between 12 to 17mg/dl).One standard unit of blood (450 ml) was drawn from each of the 128 donors. 64 units for storage as unfiltered packed cell were drawn into blood bags containing 63 ml citrate, phosphate, dextrose and adenine solution (CPDA1) as anticoagulant. Units were centrifuged at 1200 G/7 min and, by using the top and bottom system, separated into plasma, buffy coat and red cell suspension. Other 64 units for storage as filtrated packed cell were drawn into triple blood bags (Leufoflex LFP-1). 

Filtration was accomplished at room temperature for units maintained at room temperature, and they were filtered within 8 hours of collection. The unfiltered whole blood units were mixed several times by inverting the collection bag #1 (pre-filter bag), several times and the collection bag #1was hanged and the filtration set extended to full length. The filter was vertical, and all tubing was freely suspended without kinks. The CLlKTIP was broken at the outlet of the collection bag #1, and filtration ended when the filter had emptied and the housing had collapsed onto the filter pads. Filtered whole blood units were centrifuged to separate red cells from plasma, and the CLlKTIP of primary bag #2 was broken and leukocytes-reduced plasma transferred into satellite bag #3 and tubing transfer of satellite bag was clamped. The CLlKT1P of OPTlSOL solution bag #4 was broken and contents drained into primary bag #2 containing red blood cells. Tubing of primary bag sealed in two places, and between seals cut and separated from satellite bag(s).

One hour after preparation, level of electrolytes Sodium, Potassium, and Calcium(Na, K, and Ca) of serum in all blood bags were measured when we could compared level of electrolytes in both groups. Measuring of Na and K was performed by Flaim Photometer Chroning C-460 and Calcium level was measured by Auto-analyzer Ra 1000 technicon. Hemoglobin concentration of all blood bags were obtained by SYSMEX (Hematology Analyzer sysmex KX-21).

**Statistical Analysis**

After gathering data, they were analyzed by SPSS 16.5 for Windows with using of Descriptive Statistics and based on Independent T-test.

## Results

Results of this study showed that mean of age in unfiltered blood bags (group 1) was 36.83+/- 9.99 mounts and in group 2was 34.25+/-9.55 mounts. Mean of age in all blood bags was 35.54+/-9.81 which there was no significant difference between 2 groups. (P-value= 0.13) 

Mean of amount of Calcium in group 1 was 6.16+/-1.09 mg/dl and in group 2, 5.57+/-2.21 mg/dl (P-value=0.055).Also Mean of amount of Sodium in group 1 was 155.91+/-9.51 meq/l and in group 2, 153.05+/-13.21 meq/l (P-value= 0.163). As it was a distinctive study, there is no significant difference between two groups for amount of Calcium and Sodium.

However, our results showed significant difference in amount of Potassium in 2 groups with P- value<0.001 (Table I).

There is significant difference between 2 groups for hemoglobin concentration. Mean of hemoglobin concentration in group 1 was 13.86+/-2.19g/dl and in group 2, 23.16+/-3.22 g/dl (P-value <0.001). 

**Figure1:**
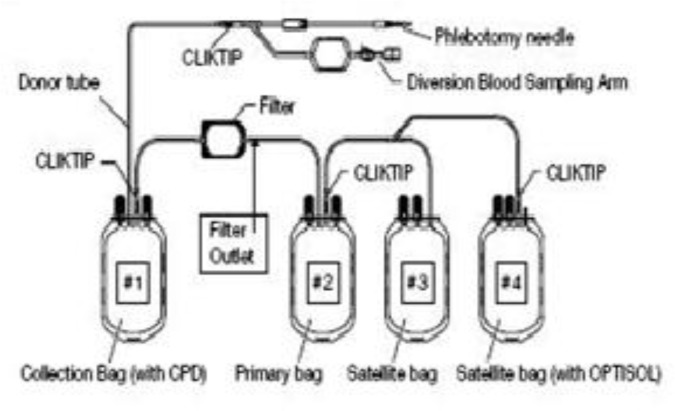
This figure shows different units of a filtered bag

**Table I:** Mean of electrolytes in unfiltered and filtered units

	**Total**		**filtered packed cell**		**unfiltered packed cell**		**Group** ** variable**
**P-value**	**SD**	**Mean**	**SD**	**Mean**	**SD**	**Mean**	
**<0.001**	2.43	6.21	2.45	7.42	1.72	5.01	**K (meq/l) **
**<** **0.055**	1/67	5/87	2.21	5.56	1.09	6.16	**Ca (mg/dl)**
**<** **0.163**	11/55	154/48	13.21	153.05	9.51	155.91	**Na (meq/l)**

## Discussion

White blood cells are present in all cellular blood components that are prepared by standard techniques. Studies have increasingly shown that leukocytes in erythrocyte and platelet preparations can lead to serious morbidity and even mortality in at-risk recipients, and cause so many undesirable effects after their transfusion (1). This is particularly so in those who require prolonged blood product support such as patients with thalassemia. Examples of adverse effects of leukocyte contamination are anaphylactic shock, febrile non hemolytic transfusion reactions (FNHTR), refractoriness to platelet transfusions, transfusion associated graft versus host disease (TAGVHD), and the transmission of infectious agents. Leukodepletion is the best method of preventing or delaying such effects. A leukocyte-reduced component is defined as containing fewer than 5 x 10^6^ white blood cells per unit of component. Leukocyte removal filters frequently have been used to produce leukocyte –reduced blood products. The filtration can be performed either at the bedside during transfusion or prior to storage. Prestorage leukoreduction will minimize the transfusion of potentially immunogenic blood cell (WBC) fragments, which accumulate during storage and may not be removed by filtration (12).

However the side effect of leukocyte depletion by filter is not completely understood.

It was hypothesized the generation of a large amount of bradykinin by filtration of PCs through a negatively charged filter might cause hypotensive reactions in patients (13, 14, 15). In a study, Masayuki et al showed bradykinin formation in red blood cell concentrates upon prolonged storage (16). After filtration of the red blood cell concentrates through negatively charged filters, they observed additional bradykinin. However hypotensive reactions are rare in patients receiving red blood cell transfusions, and they concluded that bradykinin is not likely to be the main cause of hypotensive reactions. These data, in conjunction with the reports of Bo¨nner et al and Takahashi et al, suggested that bradykinin, even if present in platelet concentrates, is not responsible for the hypotension that is associated with transfusion reactions (17, 18).

We evaluated changes of electrolytes in prestorage LR of RBCs. Similar to study of E. ERGUL EKİZ and coworkers and other studies (19, 5, and 16), in our study filtered packed cell had higher average K, but mean of Na did not change in this study. These electrolytes changes are probably due to storage-induced ATP depletion, which affects the membrane transport mechanisms (5, 15). Thus the Na/K membrane pumps start to fail, and intracellular K leaks into the storage media (22, 23).

Filtration can facilitate allocation of electrolytes. There are not other literatures reported evidences about changes of Cain prestorage LR of RBCs.

Similar to finding of E. ERGUL EKİZ and coworkers, our finding showed Hb levels were significantly higher in the LR units than in the unfiltered units. This might be the result of sedimentation that occurred during the filtration and preparation of the packed cell units (20, 21).During filtration, the sedimentation that occurred in the primary bag, which contained the unfiltered RBCs, caused the transition of more RBCs to the empty satellite bag, which contained the LR packed cell unit.

However, this effect has not been uniformly observed, particularly in studies that enrolled relatively small patient numbers.

## Conclusion

Leukocyte contamination during blood transfusion can cause many adverse effects. Prestorage leukoreduction may prevent or ameliorate some of these harmful effects, but these data suggested that prestorage leukocyte depletion by filter can change amount of electrolytes and hemoglobin concentration in packed cells.
